# Is there an association between fibromyalgia and below-normal levels of urinary cortisol?

**DOI:** 10.1186/1756-0500-1-134

**Published:** 2008-12-22

**Authors:** Silvia Izquierdo–Álvarez, Julia Pilar Bocos–Terraz, Jose Luis Bancalero–Flores, Lenin Pavón–Romero, Enrique Serrano–Ostariz, Cayetano Alegre de Miquel

**Affiliations:** 1 Department of Biochemistry (Hormonal Section), Hospital Universitario Miguel Servet, Zaragoza, Spain; 2 Department of Psychoimmunology, Instituto Nacional de Psiquiatría Dr Ramón de la Fuente Muñiz, Mexico; 3 Department of Physical Activity Medicine, Universidad de Zaragoza, Spain; 4 Rheumatology Section, Hospital Vall de Hebrón, Barcelona, Spain

## Abstract

**Background:**

Adynamia in fibromyalgia (FM) may be an expression of a functional deficit of the hypothalamic-pituitary-adrenal axis and be associated with below-normal levels of urinary cortisol. Our aim was to demonstrate that urinary cortisol was lower in patients with FM than in healthy subjects.

**Findings:**

We measured urinary cortisol levels for a sample of 47 women aged 29 to 64 years (mean age 53 years), diagnosed with FM 2–3 years previously, and compared the results with those for a control sample of 58 healthy women of a similar age. Samples of 24-hour urine were appropriately collected and levels of urinary cortisol were measured using the fluorescence polarization immunoassay method. The mean cortisol value for the women with FM was 65.40 ± 27.10 μg/L, significantly lower than the mean cortisol level for the control group, at 90.83 ± 38.17 μg/L (p < 0.001).

**Conclusion:**

Our study confirms that women with FM have significantly lower urinary cortisol levels than healthy women.

## Background

Fibromyalgia (FM) is an illness of unknown etiology that is characterized by chronic pain that patients locate in the musculoskeletal system [1]. Tending to develop from around the age of 45–55 years, mainly in women, it affects between 2% and 4% of the world population. In Spain, maximum prevalence (4.9%) occurs among adults aged 40–49 years old [1].

FM is influenced by factors such as stress, illness, and pain in some – but not all patients – as well as by certain neurotransmitter and neuroendocrine changes, including reduced biogenic amine levels, increased excitatory neurotransmitter concentrations and alterations in the hypothalamic-pituitary-adrenal axis and in autonomic nervous system activity [2-4]. Studies of cortisol levels and the hypothalamic-pituitary-adrenal axis are motivated by the high prevalence of stress and depression reported for patients with FM.

Abnormalities are not generally detected in laboratory tests unless the patient is being tested for another illness. Factors that contribute to the development or perpetuation of FM include endocrine alterations [5]. Studies have demonstrated a latent decrease in the level of certain hormones, such as the growth hormone, prolactin, adrenocorticotrophic hormone and cortisol [6,7].

Growth hormone and cortisol deficiencies lead to multifunctional disorders in response to stress, with the decrease in adrenocortical feedback possibly explaining the intense and permanent exhaustion experienced by patients with FM. Adrenal production of cortisol is also found to be lower in patients with FM, and the cortisol deficit, peripheral in origin, combines with hypothalamic-pituitary-adrenal axis dysfunctioning; the same happens with the growth hormone and insulin-like growth factor 1 [8,9]. Low cortisol levels cause reduced adrenalin production, leading to defective organic response to stress. It has also been demonstrated that patients with FM show greater resistance to the production of cortisol. A lack of cortisol can generate reduced tolerance to exertion and greater muscular fatigue and is also associated with mood swings, sleep disorders and depression [10,11].

Some authors suggest that the combined reduction in cortisol, prolactin and GH levels could be a good biochemical marker of FM [12].

No specific laboratory test exists for FM. However, it is important to study the existence of other clinical manifestations that could produce similar symptoms or complicate the course of the FM. Recommended tests include a complete blood count, erythrocyte sedimentation rate, creatine kinase, C-reactive protein, thyrotropin and protein profile [1,13]. Most studies to date have fundamentally focused on studying varying cortisol levels in saliva and blood, but have failed to arrive at conclusions as to why variations occur in patients with FM. Reduced levels of cortisol in the blood of patients with FM have not been reported; on the contrary, in some cases, considerable increases have been reported [14].

Given that determining cortisol is not relevant from a clinical point of view and also that urinary cortisol is a reflection of the free fraction of the hormone, urinary cortisol is more representative of organic secretion than plasma cortisol.

We conducted a study of urinary cortisol levels for a group of women diagnosed with long-standing FM (2–3 years) and compared these levels with those for a group of healthy women of a similar age. The aim of this study was to verify whether the urinary concentration of cortisol in the group of patients with FM was significantly lower with respect to the control group and so explain the adynamia of patients with FM.

## Methods

The study design and protocol were reviewed and approved by the Ethics Committee of the Hospital Universitario Miguel Servet in Zaragoza, Spain, in accordance with the Declaration of Helsinki and the Nuremberg Code. All the patients in the study granted their informed consent.

A sample group of 53 women was randomly selected from among members of the Aragonese Fibromyalgic and Chronic Fatigue Association (ASAFA). These women had been diagnosed in accordance with American College of Rheumatology diagnostic criteria for FM. Exclusion criteria were pregnancy, hypothyroidism and the use of corticosteroids and anti-depressive drugs in the 6 months prior to sampling, leading to the exclusion of 6 patients from the initial sample of 53 women study. The patients, aged between 29 and 64 years (mean, 53 years) and with a normal body mass index (that is, 18.50 to 24.99 kg/m^2^), had long-standing FM (2–3 years). In order to establish normality values for urinary cortisol for comparison with the values obtained for the patients with FM, urine samples were also taken for 58 healthy women in a very similar age range (30–65 years, mean 45.5), who acted as a control group.

Urinary cortisol was quantitatively determined by means of a fluorescence polarization immunoassay (FPIA) run on an AxSYM autoanalyzer (Abbott Laboratories). The analytical characteristics of the method were precision and accuracy of ≤ 5%, sensitivity of 95%, coefficient of variation of < 5% and a detection limit of ≤ 1 μg/mL. The sample was an aliquot of 24-hour urine, appropriately collected and delivered immediately and directly to the laboratory.

Samples that could not be processed in the first 24 hours were kept cool; if processing was postponed for more than 4 days, the samples were frozen at -20°C.

Statistical analyses were performed using SPSS for Windows, Version 13.0. The Kolmogorov-Smirnov test was used to determine that distributions for the FM and control groups were normal for the studied variables, namely, μg urinary cortisol/L, μg cortisol/24-hour urine (bearing in mind diuresis) and the cortisol/creatinine ratio (to evaluate whether urine sampling had been performed correctly).

For normal distributions, the *t *test for independent samples was applied; in other words, the means and variances for the two independent groups were compared. For non-normal distributions, we used the non-parametric Mann-Whitney U test, with the corresponding calculation of significance levels.

## Results

The mean 24-hour urinary cortisol value for the control group was 90.83 ± 38.17 μg/L, compared to a value of 65.40 ± 27.10 μg/L for the patients with FM (p < 0.001).

The cortisol value expressed in μg/24 hours was 144.22 ± 64.77 for the control group and 137.00 ± 201.05 for the FM group (p < 0.5). The cortisol/creatinine ratio was 116.82 ± 62.86 for the control group and 97.50 ± 38.42 for the FM group (p < 0.5).

The reference range for 24-hour urinary cortisol showed wide variation. In all probability, the absence of appropriate separation (using high-performance liquid chromatography) results in a false signal due to cross-reactivity with reduced cortisol metabolites, which are present in high concentrations in urine.

The values for 24-hour urinary cortisol depended on urine collection quality – a key factor in terms of obtaining reliable results, given that individual variations and metabolic or pharmacological interference may alter metabolism. Most of the patients with FM presented lower levels of urinary cortisol than normal, an observation that corroborates other studies [5,10,14] indicating lower adrenal production of cortisol in patients with FM. Previous studies showed urinary free cortisol levels in patients with FM to be within the normal range [14-16] or significantly below the lower normal limit [9,17].

The 24-hour urinary cortisol value depends on the analytical selectivity of the antiserum used for the immunoassay. The fact that antiserum cross-reactivity may significantly influence the respective cutoff values appears to be the major drawback of the proposed method.

The cortisol deficit found in the patients in our study could partly account for their FM and be associated with adynamia, mood swings, sleep disorders, depression and increased muscular pain following exercise [18]. FM does not improve in response to treatment with corticosteroids [19].

With regard to using 24-hour urinary cortisol levels as a possible biochemical marker for FM, treatment with corticosteroids or anti-depressants should be taken into account, given that such drugs tend to raise cortisol levels.

## Conclusion

Information on 24-hour urine cortisol levels does not constitute useful information in itself, but may be valuable when combined with other clinical tests normally performed to diagnose FM. Although a dysfunctional hypothalamic-pituitary-adrenal axis can lead to decreased sensitivity and resiliency in the stress system, it does not necessarily affect baseline cortisol levels, so 24-hour urinary cortisol levels alone cannot be used to evaluate FM. Urinary cortisol is a potentially useful biochemical marker in terms of evaluating adynamia in FM, along with insulin-like growth factor 1, substance P and the terminal telopeptides [20]. Nonetheless, studies based on larger samples are necessary in order to corroborate these conclusions.

## Competing interests

The authors declare that they have no competing interests.

## Authors' contributions

SIAcarried out laboratory tests, participated in designing the study and performed the statistical analysis. PBTcarried out the assays and participated in designing the study. JLBFparticipated in the sequence alignment and helped draft the manuscript. LPRparticipated in designing the study.ESOconceived the study, participated in its design and coordination and helped draft the manuscript. CAMhelped draft the manuscript, revised it critically for intellectual content and gave final approval of the version to be published.
